# No Increase in Short-Term Mortality Following COVID-19 Vaccination Among Nursing Home Residents

**DOI:** 10.1093/geroni/igab046.058

**Published:** 2021-12-17

**Authors:** Barbara Bardenheier, Stefan Gravenstein, Roee Gutman, Neil Sarkar, Richard Feifer, Elizabeth White, Vincent Mor

**Affiliations:** 1 Brown University School of Public Health, Smithfield, Rhode Island, United States; 2 Brown University, Providence, Rhode Island, United States; 3 Brown University, providence, Rhode Island, United States; 4 Rhode Island Quality Institute, Providence, Rhode Island, United States; 5 Genesis HealthCare, Kennett Square, Pennsylvania, United States

## Abstract

Reports of fatal adverse events following mRNA-based vaccination for COVID-19 in Norwegian nursing home (NH) residents have raised concern regarding vaccine safety in very old and frail persons. A limitation of these reports, however, is the absence of contemporaneous control groups, particularly given the high baseline mortality in this population. Using electronic health records’ data on resident deaths, hospital transfer, vaccination, and daily census from Genesis Healthcare, a large NH provider spanning 24 U.S. states, we compared 7-day mortality and hospitalization rates for vaccinated versus unvaccinated NH residents. Between December 18, 2020 and December 31, 2020, 7006 residents across 118 NHs were vaccinated with the first dose. Mortality and hospital transfer rates within 7 days of vaccination were compared to rates for: (1) unvaccinated residents in the same facility within 7 days of the vaccine clinic (n=4414), and (2) residents in 166 yet-to-be-vaccinated facilities between December 25, 2020 and January 1, 2021 (n=17,076). We excluded residents with a positive SARS-CoV-2 diagnostic test within 20 days prior to their 7-day observation window. Mortality rates per 100,000 residents were lower among vaccinated (587, 95%CI: 431, 798) versus unvaccinated residents within the same facilities (984, 95%CI: 705, 1382), and compared to residents in not-yet-vaccinated facilities (912, 95%CI: 770-1080), with overlapping 95% CIs. Hospital transfers were lower among vaccinated residents than in either comparison group, but with overlapping CIs. Our findings suggest that short term mortality rates appear unrelated to vaccination for COVID-19 in NH residents, and should dispel concerns raised by previous reports.

